# Shifting Paradigms in SCAD Care: Favoring OMT Over Intervention – Lessons from a National Cohort

**DOI:** 10.31083/RCM46760

**Published:** 2025-12-19

**Authors:** Ahmed Hegazi Abdelsamie, Hani Omar Abdelhadi

**Affiliations:** ^1^Cardiology Department, Mouwasat Hospital Dammam, 32263 Dammam, Saudi Arabia

## 1. Introduction

Spontaneous coronary artery dissection (SCAD) is a serious cause of acute coronary syndrome (ACS), yet optimal management remains debated. Krittanawong *et al*. (2024) [1] in *Reviews in Cardiovascular Medicine*, provide compelling evidence from a national cohort comparing conservative management (optimal medical therapy, OMT) versus percutaneous coronary intervention (PCI) in 31,105 SCAD patients.

SCAD is an increasing cause of ACS, especially in women without conventional cardiovascular risk factors [2]. Studies have reported that ACS in women under 50 are caused by SCAD, which is characterized by spontaneous intimal tears or intramural hematomas in coronary arteries. It is frequently linked to fibromuscular dysplasia (FMD), hormonal changes, peripartum and systemic inflammatory conditions [3]. The management of SCAD remains controversial, with discussions contrasting conservative treatment (such as beta-blockers and aspirin) with PCI, which carries a risk of complications such as the propagation of the dissection [4]. The 2024 study by Krittanawong *et al*. [1] in *Reviews in Cardiovascular Medicine* provides strong insights into in-hospital outcomes by comparing the results of OMT to PCI in 31,105 SCAD patients using the National Inpatient Sample (NIS). This commentary critically evaluates their findings, highlighting the clinical and research implications for the management of SCAD.

## 2. Critical Analysis of the Study Findings

One of the largest studies to date is the retrospective analysis conducted by Krittanawong *et al*. [1]. The study examined 31,105 SCAD patients (10,480 OMT, 20,625 PCI) between 2016 and 2020 and found that the use of PCI decreased from 72% in 2016 to 60% in 2020. The results showed that the PCI group had marginally higher mortality in propensity-matched analyses (odds ratio (OR) 1.58, *p* = 0.051) than the OMT group, with higher in-hospital mortality (OR 1.89, 95% confidence interval (CI) 1.24–2.90), cardiogenic shock (OR 2.29), and the use of mechanical circulatory support (e.g., left ventricular assisted device, OR 3.97). OMT’s benefits for stable SCAD patients are further supported by the study’s findings of increased hospitalization costs and longer hospital length of stay with PCI [1].

Among the study’s advantages are its nationwide database and increased sample size, which offer strong statistical power for less common events (mortality: 1.62% OMT vs. 5.67% PCI) [1]. Despite the PCI group’s increased age (mean 64 vs. 54), propensity-score matching reduced confounding by balancing comorbidities including diabetes and hypertension (79% PCI vs. 59% OMT) [1]. Because of the possibility of spontaneous healing, the results support the recommendation of Hassan *et al*. [5] in 2021 for conservative treatment of stable SCAD to avoid post interventional complications such as antegrade or retrograde propagation of the dissection, arterial perforation, and stent thrombosis. The study’s temporal trend data supports the premise that the observed decrease in PCI is a result of increased knowledge of these recommendations.

There are, however, limitations which moderate the results. Granular information that is essential for determining the appropriateness of an intervention, such as PCI indications (e.g., left main (LM) involvement, continuous ischemia) or angiographic characteristics (e.g., dissection length, thrombolysis in myocardial infarction (TIMI) flow grade), are absent from the NIS’s retrospective design [6]. Since more high risk patients probably needed PCI for unstable presentations, the higher comorbidity burden in the PCI group raises the possibility of selection bias [7]. The marginal mortality significance (*p* = 0.051) following propensity matching, raises concerns about clinical heterogeneity and statistical power, possibly as a result of unmeasured variables [1]. Since the diagnosis of SCAD is dependent on the identification of specific angiographic patterns [8], Pender *et al*. [9] suggest that the low prevalence of FMD (0.15–2.43% vs. 25–86% in literature) might be the result of inconsistent screening or inaccurate International Classification of Diseases, Tenth Revision, Clinical Modification (ICD-10-CM) (I25.42) classification.

The cohort’s low diversity (66–73% White) differs from Pender *et al*.’s [9] broader ethnic data (e.g., 5% Māori in ANZ Registry), limiting its applicability to minority communities. Finally, Dang *et al*. (2025) [10] point out that the emphasis on in-hospital outcomes leaves out long-term recurrence data (10–20% risk). They demonstrate that SCAD cannot be regarded as a benign condition because it is associated with a significant risk of major adverse cardiovascular events (MACE) and an increased incidence of recurrent episodes of SCAD. The increased risk of MACE and recurrence is linked to the high prevalence of FMD or other extra-cardiac vascular abnormalities in patients with SCAD [10].

In contrast, Pender *et al*.’s (2025) [9] study reported the prevalence of SCAD at 1–4% of ACS cases, with a mean age of 51 and an 80–90% female predominance. This suggests that the older cohort (mean 54–64) in Krittanawong *et al*.’s [1] study may not accurately reflect normal SCAD demographics. In addition, Pender *et al*. [9] draw attention to the severity of pregnancy-related SCAD (e.g., 24% left main involvement), which Krittanawong *et al*. [1] do not discuss. In contrast to Krittanawong *et al*.’s [1] preference for OMT in more general SCAD cases, Morosato *et al*. [11] on LM SCAD (132 patients, 80% female) report higher morbidity (22% cardiogenic shock) and show benefits from early revascularization (adjusted hazard ratio (HR) 0.37 for the composite endpoint). These comparisons underscore the need for tailored management based on the location and severity of SCAD.

## 3. Clinical Implications

Krittanawong *et al*.’s [1] findings suggest conservative management as the recommended treatment for the majority of SCAD patients, particularly those without hemodynamic instability, due to the lower mortality and morbidity associated with OMT. According to Morosato *et al*.’s [11] findings on LM SCAD, clinicians should prioritize OMT (e.g., beta-blockers, aspirin) for stable SCAD and save PCI for high-risk presentations, including left main dissection. For example, a 45-year-old woman with SCAD who presents with chest discomfort but stable vitals should undergo coronary angiography, followed by intravascular ultrasonography (IVUS) or optical coherence tomography (OCT) if available, and then OMT unless the symptoms worsen. In contrast, the study by Morosato *et al*. [11] suggests that early revascularization is necessary for LM SCAD with cardiogenic shock in order to minimize adverse outcomes.

According to Pender *et al*. [9], young women with SCAD report feeling anxious and concerned about recurrence, which makes multidisciplinary care (psychologists, support groups, and cardiologists) necessary. Given their importance to patient recovery, Krittanawong *et al*.’s [1] absence of psychosocial outcomes is a significant limitation [9]. Since FMD is highly prevalent in SCAD, as reported by Pender *et al*. [9], screening for FMD and connective tissue disorders (such as Marfan syndrome) using magnetic resonance angiography is essential, as suggested by Fatrous *et al*. [8] (Table 1, Ref. [1, 9, 10, 11]).

**Table 1.** **Summarizes clinical recommendations based on Krittanawong *et al*. [1] and current studies**.

Aspect	Recommendation	Reference
Diagnosis	Use coronary angiography; supplement with IVUS/OCT if available	Krittanawong *et al*., 2024 [1]
Risk Factors	Screen for FMD, hormonal triggers, and connective tissue disorders	Pender *et al*., 2025 [9]
Management	Prioritize conservative therapy (beta-blockers, aspirin); consider PCI for high-risk cases	Krittanawong *et al*., 2024 [1] Morosato *et al*., 2025 [11]
Psychosocial Care	Offer counseling and support groups to address anxiety and recurrence fears	Pender *et al*., 2025 [9]
Follow-Up	Monitor for recurrence with regular clinical assessments, including imaging and biomarker evaluation	Dang *et al*., 2025 [10]

IVUS, intravascular ultrasonography; OCT, optical coherence tomography; FMD, fibromuscular dysplasia; PCI, percutaneous coronary intervention.

## 4. Research Implications

The observational design of Krittanawong *et al*. [1] cannot adequately address causality due to selection bias, necessitating randomized controlled trials (RCTs) to compare OMT with PCI, particularly for high-risk subsets such as LM SCAD, as highlighted by Morosato *et al*. [11] and Adlam *et al*. [12]. The minimal amount of diversity in Krittanawong *et al*’s [1] study cohort contrasts with Pender *et al*.’s [9] request for worldwide registries to include underrepresented groups (for example, Aboriginal populations). To better assess the risk recurrence, long-term follow-up studies are essential, such as the study by Dang *et al*. (2025) [10], which found a 5.7% incidence of recurrence at three years. Mechanistic studies into hormonal triggers (for example, estrogen’s function in arterial fragility) and biomarkers will improve risk stratification, particularly for pregnancy-related SCAD [9, 13].

The majority of SCAD patients have ischemia-related changes, as demonstrated in case series that examined the cardiac computed tomography (CT) and cardiac magnetic resonance imaging (MRI) results in these individuals. A small percentage of patients had no anomalies. Non-invasive imaging can be a useful tool for diagnosing SCAD in stable individuals instead of more invasive procedures that entail a higher risk of complications [3].

Developing SCAD-specific registries, funded by organizations such as the American College of Cardiology, could provide longitudinal data to influence guidelines, which are still inadequate when compared to atherosclerotic disease. Non-invasive imaging, such as coronary CT angiography and Cardiac MRI, should be investigated to improve diagnosis in areas with limited resources. Finally, given the chronic nature of SCAD, patient-centered outcomes such as quality of life and mental health require further attention.

## 5. Conclusion

Krittanawong *et al*.’s [1] study provides strong evidence that conservative therapy with OMT is preferable to PCI for the majority of SCAD patients, resulting in lower death and complication rates. Compared to Pender *et al*.’s [9] epidemiological insights and Morosato *et al*.’s [11] findings on LM SCAD, it emphasizes the importance of individualized therapies based on the severity of symptoms at the time of presentation. Clinicians should prioritize OMT, sophisticated imaging, and psychosocial therapy for stable SCAD, with revascularization reserved for high-risk patients. To optimize SCAD management and improve results for young females and larger populations, researchers must conduct RCTs involving multi-national registries and mechanistic studies.

## Abbreviations

ACS, acute coronary syndrome; CI, confidence interval; FMD, fibromuscular dysplasia; HR, hazard ratio; IVUS, intravascular ultrasonography; LM, left main; MACE, major adverse cardiovascular events; OR, odds ratio; OCT, optical coherence tomography; OMT, optimal medical therapy; PCI, percutaneous coronary intervention; RCTs, randomized controlled trials; SCAD, spontaneous coronary artery dissection.
